# Validity of a two-antibody testing algorithm for mismatch repair deficiency testing in cancer; a systematic literature review and meta-analysis

**DOI:** 10.1038/s41379-022-01149-w

**Published:** 2022-09-14

**Authors:** K. T. S. Aiyer, T. Doeleman, N. A. Ryan, M. Nielsen, E. J. Crosbie, V. T. H. B. M. Smit, H. Morreau, J. J. Goeman, T. Bosse

**Affiliations:** 1 Department of Pathology, Leiden University Medical Center, PO Box 9600, 2300 RC Leiden, Leiden, The Netherlands; 2 Division of Cancer Sciences, Faculty of Biology, Medicine and Health, University of Manchester, St Mary’s Hospital, Manchester, UK; 3 The Academic Women’s Health Unit, Translational Health Sciences, Bristol Medical School, University of Bristol, Bristol, UK; 4 Department of Clinical Genetics, Leiden University Medical Center, PO Box 9600, 2300 RC Leiden, Leiden, The Netherlands; 5 Department of Obstetrics and Gynecology, Manchester Academic Health Science Centre, Manchester University NHS Foundation Trust, Manchester, UK; 6 Department of Biomedical Data Sciences, Leiden University Medical Center, PO Box 9600, 2300 RC Leiden, Leiden, The Netherlands

## Abstract

Reflex mismatch repair immunohistochemistry (MMR IHC) testing for MLH1, PMS2, MSH2 and MSH6 is used to screen for Lynch syndrome. Recently MMR-deficiency (MMRd) has been approved as a pan-cancer predictive biomarker for checkpoint inhibitor therapy, leading to a vast increase in the use of MMR IHC in clinical practice. We explored whether immunohistochemical staining with PMS2 and MSH6 can be used as a reliable substitute. This two-antibody testing algorithm has the benefit of saving tissue, cutting costs and saving time. PubMed, Embase and Cochrane library were systematically searched for articles reporting on MMR IHC. The weighed percentage of cases with isolated MLH1 or MSH2 loss or combined MLH1/MSH2 loss alone was analyzed using a random effects model meta-analysis in R. The search yielded 1704 unique citations, of which 131 studies were included, describing 9014 patients. A weighed percentage of 1.1% (95% CI 0.53–18.87, I = 87%) of cases with isolated MLH1 or MSH2 loss or combined MLH1/MSH2 loss alone was observed. In the six articles with the main aim of investigating the two-antibody testing algorithm all MMRd cases were detected with the two-antibody testing algorithm, there were no cases with isolated MLH1 or MSH2 loss or combined MLH1/MSH2 loss alone. This high detection rate of MMRd of the two-antibody testing algorithm supports its use in clinical practice by specialized pathologists. Staining of all four antibodies should remain the standard in cases with equivocal results of the two-antibody testing algorithm. Finally, educational sessions in which staining pattern pitfalls are discussed will continue to be important.

## Introduction

Mismatch repair deficiency (MMRd) occurs in ~10–15% of colorectal carcinomas (CRC) and ~25–30% endometrial carcinomas (EC)^1–10^. MMRd can be caused by somatic *MLH1* promotor hypermethylation, somatic mutations combined with loss of heterozygosity or bi-allelic mutations. Furthermore, MMRd can also result from germline mutations (Lynch syndrome) in mismatch repair (MMR) genes in combination with a second hit on the wildtype allele^11^. Lynch syndrome accounts for ~20% of MMR-deficient CRC and ~10% of EC^1,3,6,12–15^. Many professional societies have recommended reflex MMR testing for CRC and EC by initial MLH1, PMS2, MSH2 and MSH6 IHC to triage for subsequent germline testing. Subsequent *MLH1* promotor hypermethylation or BRAF testing is performed in cases with MLH1/PMS2 loss^16–21^. Recent analyses have shown this pre-screening approach for germline testing to be cost effective^22–30^. In addition, MMR testing is not limited to CRC and EC. Patients with other types of Lynch syndrome associated carcinomas (e.g., ovarian, stomach and urothelial carcinomas) receive MMR IHC on indication, such as a positive family history of Lynch syndrome or a personal history of Lynch syndrome associated carcinomas^31^.

MMRd testing is no longer simply a matter of screening for individuals who have undiagnosed Lynch syndrome. Patients with cancer types with high mutational burden, specifically MMRd colorectal, endometrial, gastric, bladder, breast, ovarian, bile duct/gall bladder, pancreatic, small cell lung and thyroid carcinomas have been shown to benefit from checkpoint inhibition^32–36^. As a result, MMRd has been approved by the FDA as a pan-cancer predictive biomarker for checkpoint inhibition benefit. Therefore, correctly identifying cancers with MMRd enables targeted and effective treatment with checkpoint inhibitors in addition to Lynch syndrome screening.

MMRd, microsatellite instability (MSI), tumor mutational burden (TMB) and programmed death-ligand 1 (PD-L1) IHC have all been recognized as predictive biomarkers of the efficacy of checkpoint inhibition^37–40^. Next generation sequencing (NGS) can be used to index MSI or TMB^41–43^. MSI can also be objectified by specific polymerase chain reaction (PCR) testing with a panel of microsatellite markers. Both NGS and MSI testing require DNA isolation from the tumor, are costly and take at least a few days to generate a result^44,45^. As MMR IHC is a sensitive, relatively fast and cheap method to identify MMRd, it is frequently put forward as the preferred test^12^. In contrast to MSI testing, MMR IHC provides information with regard to the involved gene. Furthermore, it may identify cases with subclonal loss of expression (loss of expression in a specific area of the tumor), which may be missed by localized DNA extractions. Other benefits include the long-term experience pathologists have with evaluating MMR proteins (specifically in the context of screening for Lynch syndrome) and the very little material needed for adequate evaluation. The rise in checkpoint inhibitor therapy has led to an increase of the number of MMR IHC tests performed in routine diagnostic pathology.

**Fig. 1:**
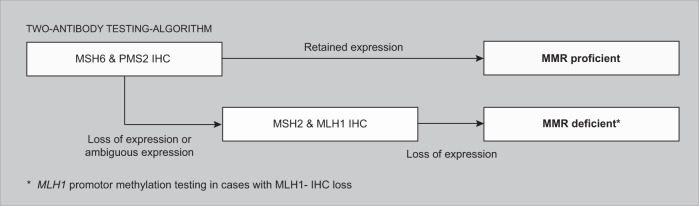
Two-antibody testing-algorithm.

The mismatch repair proteins function as two heterodimer complexes; PMS2 forms a stable heterodimer with MLH1, while MSH6 dimerizes with MSH2. If either PSM2 or MSH6 loses protein function MLH1 and MSH2 can form a heterodimer with another protein, e.g., PMS1, MLH3 or MSH3, resulting in retained or slightly diminished immunohistochemical expression of MLH1 and MSH2. However, PMS2 and MSH6 are not able to form alternative heterodimers resulting in loss of function of the entire heterodimer complex^11^. Subsequently MMR protein expression as objectified with IHC is expected to be negative in both MMR proteins of the affected heterodimer. This knowledge forms the biological rationale for a two-antibody MMR IHC screening which is limited to immunostaining for PMS2 and MSH6, as proposed by Shia et al.^46^. The two-antibody testing algorithm may be an attractive alternative approach for reflex MMR status testing as compared to testing all four MMR proteins simultaneously (Fig. 1).

The two-antibody testing algorithm has the advantage of not having to perform four immunostainings in all cases, as MLH1 and MSH2 IHC are only performed when PMS2 or MSH6 IHC results show loss of staining or an abnormal expression pattern such as subclonal loss of staining, weak staining or uninterpretable/unusual staining. This will lead to a significant reduction in the number of slides that need to be stained and interpreted, resulting in a shorter overall processing and reporting time. Secondly, more tissue will be available for molecular analysis that might be performed later. In addition, the overall costs for these procedures will be reduced by 50% in all MMR proficient cases (MMRp), while only causing a minimal delay in MMR status assignment in MMRd. With the increasing demand for MMR status assignment these benefits are of great interest.

Several recent studies have investigated the validity of the two-antibody testing algorithm with somewhat conflicting results. Where most study results support and recommend a two-antibody testing algorithm^46–50^, one recent study of a cohort of CRC challenged its use as it observed cases with complete loss of MSH2 expression in combination with heterogeneous staining of MSH6^51^. Most guidelines prescribe the use of four antibodies in the assessment of MMR status; however, this recommendation holds little scientific basis^18,20,52,53^.

To obtain a broad view on the worldwide use and reporting of MMR IHC staining patterns we aimed to include a high number of cases from different specimen types in a systematic review of the literature. To objectify data across studies we performed a meta-analysis. The effectiveness of the two-antibody testing algorithm for the determination of MMR status assignment was assessed by calculation of a weighed percentage of MMRd cases that would not have been identified by this approach (cases with either isolated loss of MLH1/MSH2 or double loss of MLH1 and MSH2), in order to provide a scientific basis for guideline recommendations.

## Materials and methods

This systematic review was conducted and reported according to the Preferred Reporting Items for Systematic Reviews and Meta-analyses (PRISMA) guidelines^54^.

### Search strategy

A computerized literature search of PubMed, Embase and Cochrane library was conducted for all peer-reviewed studies that reported on MMR IHC in humans. The search strategy was devised in collaboration with an information specialist and consisted of a combination of Medical Subject Headings (MESH terms) and free text words with the following combined keywords: ‘immunohistochemistry’ and ‘mismatch repair’ including all relevant keyword variations. The original search strategy can be found in Supplementary Information 1 (S1). The search was performed in April 2020. Articles were limited to English language and publication dates after June 2007 due to the introduction of automated staining technology and more representative reporting patterns. The references of primary selected studies were scrutinized for additional records that were not identified through database search.

### Eligibility criteria

Two reviewers, senior pathology residents (KA &TD), independently selected and identified the appropriate studies based on prespecified inclusion and exclusion criteria using the online tool Rayyan^55^. Where consensus could not be reached, the senior pathologist (TB) made the final decision. Articles were eligible for inclusion when a detailed description of all four MMR IHC staining patterns for each individual lesion was reported and the combination of MMR IHC results were unequivocal to the reviewers. Exclusion criteria were: reviews and meta-analyses containing no original data, case reports, non-human studies and studies with insufficient data for analysis (reporting immunohistochemistry results on <20 cases).

### Risk of bias assessment

The quality of the selected studies was rated based on the quality of the immunohistochemical staining process and interpretation. Adequateness of IHC staining and interpretation was determined for each article based on the following quality parameters: use of whole slides, reporting a clear definition of MMR IHC loss, use of NORDIQC approved immunohistochemical stains, evaluation by ≥2 evaluators and evaluation by at least one pathologist. A quality score between 0 and 5 was given according to how many parameters were met. Articles meeting 4 or 5 of the quality parameters were considered to be of high quality.

The percentage of MMRd was expected to vary among the articles, due to biological differences in MMRd prevalence in cancer types and different inclusion criteria used (e.g., some studies only included MSI-high specimens). To avoid selection (sampling) bias the analysis was performed only on the MMRd cases, MMRp samples were not included in the analysis.

### Data extraction

The following information was extracted: authors, lesion type, specimen type, hospital(s) and country/countries of specimen inclusion, dates between which patients were included, use of whole slides or tissue microarray (TMA), definition of loss of MMR IHC staining used, antibody clones used for MMR IHC, type of IHC evaluator (pathologists yes/no), number of evaluators, if the aim of the study was to test the validity of the two-antibody testing algorithm compared to the traditional four antibody approach (yes/no), number of specimens with IHC reported for all 4 MMR proteins and number of MMRd specimens. The number of MMRd specimens was further specified as follows: number of specimens with isolated loss of PMS2, MLH1, MSH2 or MSH6, number of specimens with dual loss of PMS2/MLH1, MSH2/MSH6, PMS2/MSH6, PMS2/MSH2, MSH6/MLH1 or MLH1/MSH2, the number of cases with loss of 3 or 4 MMR proteins and their combination. In the event MMR status interpretation was adjusted after addition molecular tests, only the original stain scores (raw IHC data) were collected.

Cases in which the terms “subclonal”/“heterogeneous”/“focal”/“patchy” were used to describe the MMR IHC results were considered to have loss of MMR expression if the description was further clarified as an abrupt loss of nuclear MMR protein expression in clearly demarcated tumor areas with positive internal control. Cases that were reported to show “weak” or “equivocal” MMR staining were excluded, as this pattern is most likely due to artefactual loss (e.g., fixation artefact). Owing to many variables between country of inclusion of specimen cohort and specimen type, country of inclusion of specimen cohort was categorized in 5 groups (USA/Canada, Europe/Scandinavia, Asia/Middle East, Australia/New Zealand and other) and specimen type was categorized in 4 organ system groups (Gastro-intestinal (GI), Gynecological (GYN), Dermatological (DERM) and Other).

### Article selection

Articles reporting on the same specimen cohort were identified by using a combination of hospital(s) and country/countries of specimen inclusion, dates between which patients were included, lesion type, specimen type and authors. For articles with overlapping cohorts the article that met most quality parameters was included. If all articles met the same amount of quality parameters, the article with the most MMRd cases was selected.

### Data analysis and statistics

Our primary outcome was defined as the proportion of cases that would not have been identified as MMRd by the two-antibody testing algorithm, which uses PMS2 and MSH6 staining only, but showed either individual loss of MLH1/MSH2 or combined loss of MLH1 and MSH2 when using the four antibody approach. Henceforward these cases will be called non-dimeric loss cases, as the staining pattern cannot be explained by the heterodimeric function of the MMR proteins. Our secondary outcomes were the proportions of cases with non-dimeric loss in specific organ systems. The proportions of interest were calculated from the relevant numerator and denominator and were derived using a random effects model (arcsine regression). Proportions were presented along with 95% confidence intervals. To account for the differences in sample sizes between studies, a weighted mean of the estimates from each study was used. The following independent study variables were included in the meta-analysis: number of specimens with MMR IHC identified MMRd and number of cases with MMR IHC loss that would not have been identified by the two-antibody testing algorithm (single isolated loss of MLH1 or MSH2 and dual loss of MLH1 and MSH2). The sensivitity of the two antibody approach to identify MMRd compared to the gold standard of the four antibody approach could not be calculated as MMRp cases were excluded. A chi-square test was used to identify differences in the distribution of variables among categorized subgroups (e.g., specimen type, high/low quality articles).

Statistical analyses were performed in R statistical software, using the metafor package (version 4.0.3) and IBM SPSS Statistics (version 25). Forest plots were produced in R, showing the individual study results and weighted estimates together with 95% CI. The degree of heterogeneity across articles was examined using visual inspection of data and the I^2^ statistic^56^. The R data script can be found in Supplementary Information 2 (S2).

## Results

### Article selection

**Fig. 2:**
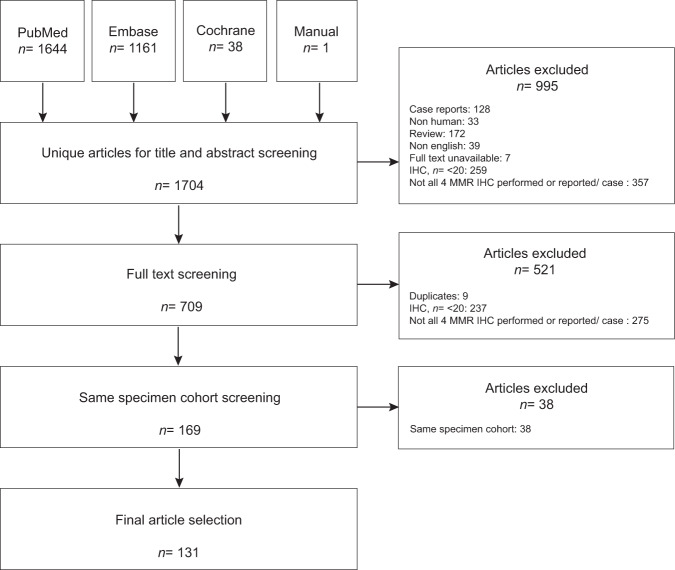
Flowchart article selection.

The literature search yielded a total of 1704 original articles (PubMed 1644, Embase 1161, Cochrane library 38, manual 1). After screening titles and abstracts, 995 articles were excluded. Seven hundred and nine articles were eligible for full-text evaluation, 559 were excluded for various reasons, and 131 articles were included in the meta-analysis (Fig. 2).

### Data distribution

Of the 131 included articles, 78 focused on gastro-intestinal cancers, 39 on cancer of the female genital tract, 6 on dermatological lesions, 4 on mixed cohorts and 4 on other specimen types (breast-, head and neck-, oral- or urological specimens) (Table 1). In total, there were 47,745 cancers of which 9014 were MMRd, varying from 20 to 661 MMRd cases per article. Non-dimeric MMR IHC patterns were reported in 298 cases. These 298 included 172 cases of isolated MLH1 loss (130 in GI, 39 in GYN and 2 in other specimen types), 114 cases of isolated MSH2 loss (88 in GI, 12 in GYN and 14 in other specimen types) and 12 cases of combined MLH1 and MSH2 loss (9 in GE and 3 in GYN) (Table 2). Cases with non-dimeric loss were reported across 40 studies. Thirteen cases, which were reported among 3 articles, were excluded due to the description of MMR IHC as “ weak” or “ equivocal”.

**Table 1:** Case distribution among specimen sites.

Specimen site	Subgroup	Articles	MMRd cases (*n* = *)*
Gastrointestinal	GI	78	5356
Gynecological	GYN	39	2758
Dermatological	DERM	6	564
Urological	Other	2	68
Oral	Other	1	21
Breast	Other	1	31
Mixed cohorts		4	216
Total		131	9014

*MMRd* mismatch repair deficiency.

**Table 2:** Distribution of MMR staining patterns.

Affected MMR Genes	IHC staining				Expected IHC pattern	Numer of cases (*n* = )
	MLH1	MSH2	MSH6	PMS2		
*MLH1*	−	+	+	−	YES	5467
*MSH2*	+	−	−	+	YES	1653
*MSH6*	+	+	−	+	YES	770
*PMS2*	+	+	+	−	YES	517
Total						8407
*MLH1*	−	+	+	+	NO	172
*MSH2*	+	−	+	+	NO	114
*MLH1* + *MSH2*	−	−	+	+	NO	12
Total						298

*MMR* mismatch repair, *IHC* immunohistochemistry.

### Risk of bias assessment

The risk of bias assessment for each article is shown in Supplementary Information 3 (S3). Twenty articles met four or five quality parameters and were considered to be of high quality. Of all the articles 51% used whole slides (*n* = 67), 63% reported a clear definition of IHC loss (*n* = 83), 19% used only NORDIQC approved immunohistochemical stains (*n* = 25), 31% were evaluated by >2 evaluators (*n* = 40) and 31% were evaluated by one or more pathologist (*n* = 41). The other articles either failed to meet the quality parameter or did not report on the given parameter. The articles with the highest percentage of unexpected MMR IHC results (isolated MLH1 or MSH2 loss or combined MLH1/MSH2 loss) are described in Supplementary Information 4 (S4).

### Percentage of isolated or combined MLH1/MSH2 IHC loss

Meta-analysis of 131 studies yielded a weighted percentage of 1.1% (95% CI 0.53–18.87, S5) for MMRd cases that were reported to show either isolated MLH1 or MSH2 loss or combined loss of MLH1 and MSH2 alone (with retained PMS2 and MSH6). Considerable heterogeneity was present (I^2^ = 87%), representing differences in results between studies. Therefore, subgroup analysis was performed.

### Subgroup analysis

**Fig. 3:**
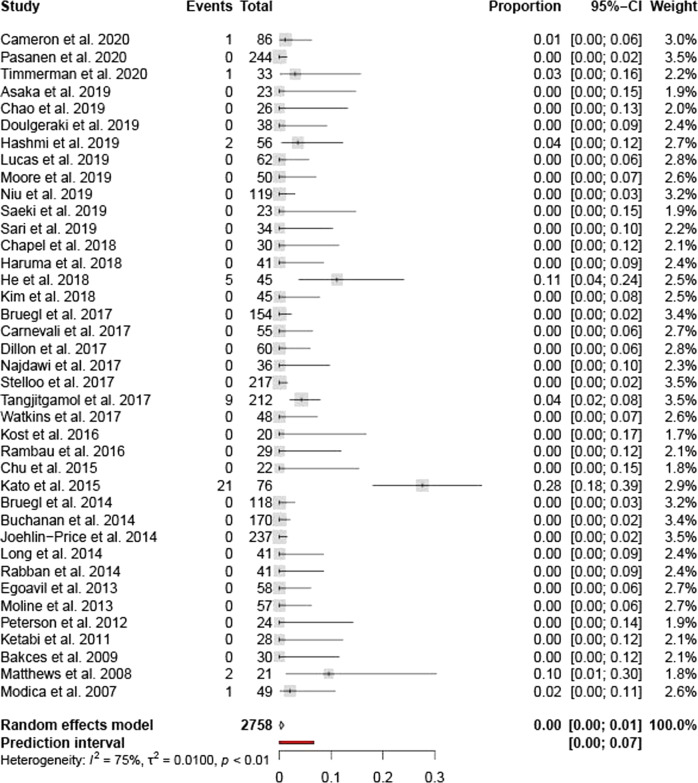
Meta-analysis forest plot showing data from all Gynecology oriented articles. The analysis included 39 articles.

**Fig. 4:**
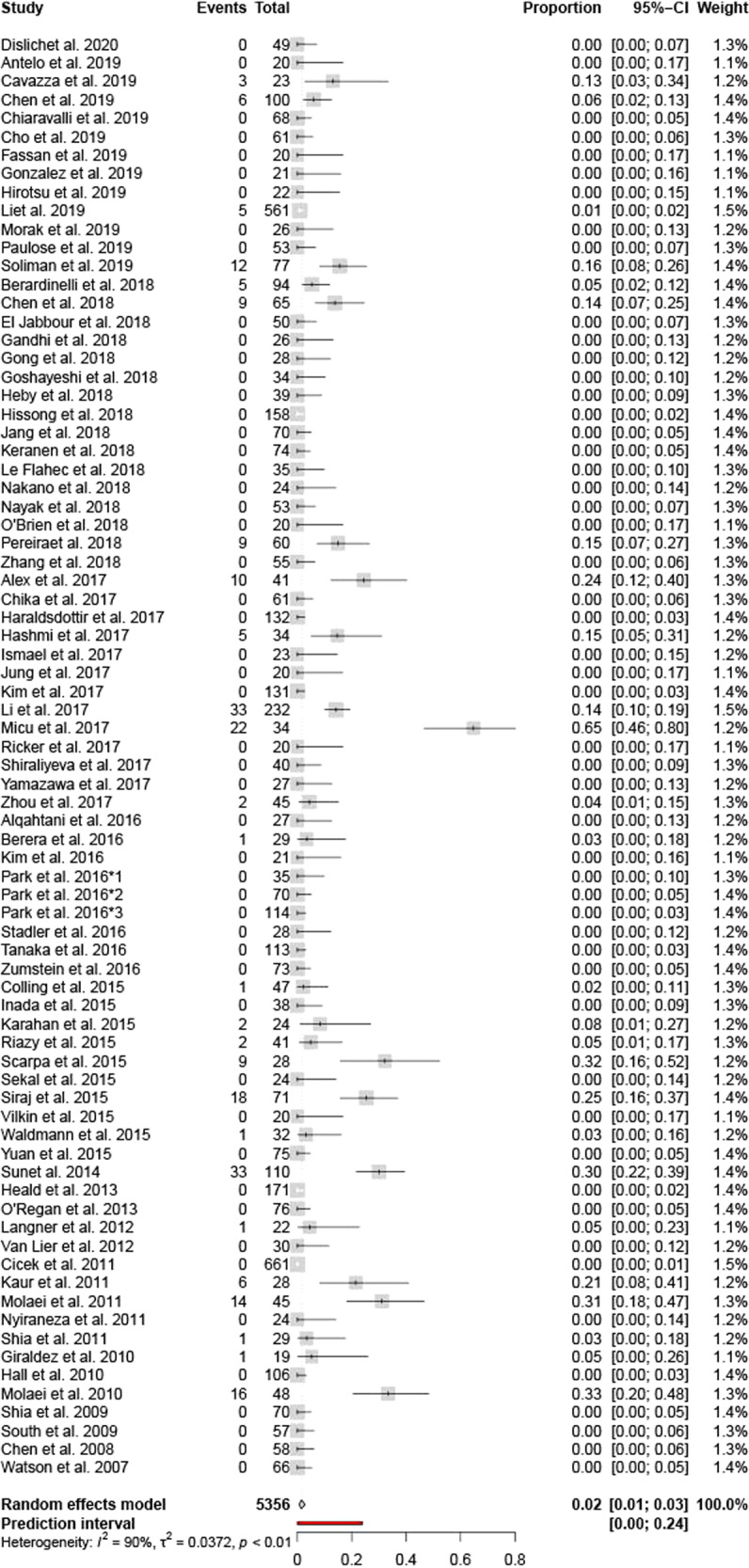
Meta-analysis forest plot showing data from all Gastrointestinal oriented articles. The analysis included 78 articles.

In six articles with the primary aim of investigating the two vs four antibody approach, no cases with non-dimeric loss were described in 505 MMRd cases (0%, CI0.00–0.00, heterogeneity I2 0%, S6)^47–51,57^. Organ system subgroup analysis showed a similarly low weighted percentage in gynaecology oriented articles and dermatology oriented articles, respectively 0.29% (95% CI 0.02-0.85, heterogeneity I^2^ 75%, Fig. 3) and 0.39% (95%CI 0.00–2.19, heterogeneity I^2^ 64%, S7). A percentage of non-dimeric loss cases of 1.54% (95%CI 0.61–2.39, heterogeneity I2 90%, Fig. 4) was observed in the articles describing lesions of the gastrointestinal tract. Heterogeneity was substantial (I^2^ > 50%) in all subgroups regarding quality score, individual quality parameters and the countries of inclusion of the specimen cohort.

### Distribution of quality parameters

Finally, we divided the articles into those without non-dimeric loss cases, articles with <5% non-dimeric loss cases and articles with >5% non-dimeric loss cases and compared these in terms of our predefined quality parameters. The group of articles without non-dimeric loss cases had a significantly higher percentage of high-quality articles (*p* < 0.05, chi-square test), compared to the ones that did report cases of non-dimeric loss. A significant difference was found for definition of nuclear loss (*p* < 0.05, chi-square test). No significant differences were found for the other individual quality parameters (pathologist scored, whole slide usage, ≥2 evaluators), publication date, cancer type, country of inclusion of specimen cohort and university/non university hospital.

### Molecular findings

Nine of the 40 articles that report non-dimeric loss cases provided some additional molecular data for these specific cases. Giraldez et al.^48^ described one case with isolated loss of MLH1 protein expression. This case was microsatellite unstable with no *MLH1* promoter hypermethylation and a pathogenic variant in *MLH1* was not identified. Timmerman et al.^49^ reported on one case with isolated MLH1 loss with confirmed hypermethylation^58,59^. Microsatellite instability was reported in 16 out of 18 cases with isolated MLH1 or MSH2 loss by Siraj et al., but subsequent testing for germline pathogenic variants or somatic *MLH1* hypermethylation was not performed. The MSH6 and PMS2 antibodies used in this study were not NORDIQC approved, and this was the only study in our meta-analysis that used the PMS2 clone C-20 by Santa Cruz Biotechnology, Dallas, Texas, USA^60^. Three cases (2x CRC and 1x skin lesion, respectively) with isolated MSH2 loss were reported to have a matching germline *MSH2* pathogenic variant. MMR IHC was re-evaluated in one of these cases and showed focal retained staining of MSH6, initially interpreted as positive^61–63^. Cavazza et al.^64^ reported on one case with isolated MLH1 loss and one with isolated MSH2 loss, with identification of germline *MSH2* pathogenic variants in both cases. In this study, the methodology of IHC staining and scoring was poorly described and no specifics were provided about the identified variants^64^. None of the articles provided images of the MMR immunohistochemical staining patterns of the non-dimeric loss cases described.

## Discussion

Reflex testing with four MMR protein immunohistochemistry for defining MMR status to triage patients at higher risk for Lynch syndrome and identify patients that may benefit from checkpoint inhibition is increasingly recommended in clinical guidelines throughout the world. The implementation of these guidelines resulted in a significant increase in MMR testing in pathology laboratories. Based on biological rationale, it has been suggested that a testing algorithm that uses just two MMR antibodies (against MSH6 and PMS2) is adequate for assessing MMR status. This is the first systematic review and meta-analysis on the performance of this two-antibody testing algorithm.

Our analysis showed a weighted percentage of 1.1% of reported cases with non-dimeric loss that would not have been identified using a two antibody approach. Considering the overall low percentage of cases with non-dimeric loss, especially in tumors of the GYN and DERM subgroups (<0.5%), implementation of the two-antibody testing algorithm seems adequate for both screening to identify patients at higher risk of having Lynch syndrome as well as identifying patients that may benefit from checkpoint inhibition therapy. The slightly higher percentage of 1.54% of non-dimeric staining patterns in the GI group is accompanied by high interstudy heterogeneity, unexplained by any of the variables (e.g., publication year, use of NORDIQC approved antibodies). Four out of 6 articles with the aim of investigating the two vs four antibody approach were GI focused and found no cases with non-dimeric loss of staining. This absence (0%) of non-dimeric staining patterns in all articles with the primary aim of investigating the two vs four antibody approach demonstrates that unexpected staining patterns will not lead to misclassification as MMRd when evaluators have their primary focus on scoring MMR IHC^47–50,57^. However, these studies were conducted by expert pathologists, and thus a four antibody approach is advisable to assure correct MMR status assigment in less experienced hands.

Issues with correct interpretation of MMR IHC staining might be a possible explanation for the reporting of non-dimeric loss of staining. Sometimes MMR protein expression is reduced in intensity, heterogeneous, focal or patchy. This is most frequently the result of inadequate fixation of tissue. In these instances the four antibody approach seems justified as the combination of stains will support the pathologist’s interpretation. The use of dated archived material can also result in lesser quality of the immunohistochemical stains. MMR staining was observed to be more intense and homogeneous in biopsies compared to resection specimens and thus easier to interpret in the former, most likely due to more uniform and complete fixation^65^. The use of biopsy material for MMR IHC is therefore preferred over the use of resection specimens. Secondary downregulation of the MMR genes should also be considered as a possible cause for unexpected staining patterns. Environmental factors such as tissue hypoxia and oxidative stress have been shown to significantly reduce the expression of MMR genes at RNA level and result in suppression of DNA mismatch repair. These factors may be caused by prolonged ischemia or delayed fixation^66^.

Another possible explanation for noncanonical staining patterns is the practice of neo-adjuvant treatment prior to MMR-assessment, this may also influence expression patterns, especially of MSH6^62,67^. As neo-adjuvant therapy in endometrial cancer is rare but common in gastro-intestinal cancers, this might partly explain the higher percentage of non-dimeric loss found in gastro-intestinal cancers. The interpretation of the MMR proteins in the context of the two-antibody testing algorithm should therefore always be evaluated with care and when in doubt about heterogeneity, focal or inequivalent staining patterns subsequent MSH2 and MLH1 should be performed. When possible, it is preferable to perform MMR IHC on (biopsy) specimens taken before neo-adjuvant therapy. Another challenge is the clinical interpretation of subclonal or regional loss of MMR expression. Subclonal loss is believed to be an acquired MMR defect arises during tumorgenesis. Whether subclonal MMR loss can be observed in a germline context requires further study^10^. The predictive value of subclonal loss for checkpoint inhibition therapy response has also not been objectified. If pathologists are aware of the staining pattern pitfalls and are able to recognize them, this should not pose a problem in routine diagnostics. The recently published paper by Gilks et al. could be used as an excellent guideline to minimize inter-observer variability and increase detection of pitfalls in staining patterns^10^.

In the literature, no biological explanation for isolated MLH1 or MSH2 loss has been reported. None of the articles that report on non-dimeric loss cases provided images and only a few report additional supporting molecular findings. A few cases with non-dimeric loss report either MSI, *MLH1* promotor hypermethylation or a germline mutation. However, these articles do not provide all information required for a complete assessment of their quality. A full molecular workup has not been performed in most articles and antibodies that are not NORDIQC approved are used. We speculate that these antibodies may have resulted in false-positive nuclear staining, consequently explaining the cases with unexpected individual MLH1 or MSH2 loss.

Using this two-antibody testing algorithm will substantially reduce costs by 50% in all MMRp cases while only causing a minimal delay in MMR status assignment in MMRd cases. Extra slides will need to be stained with MLH1 and MSH2 in MMRd cases, resulting in a reporting delay of approximately one day. We do not expect this to effect surgical planning. In contrast the number of slides that need to be stained and interpreted will be reduced by 50% in MMRp cases, resulting in shorter overall reporting time. The reduction in slides per patient may also increase accessibility in low-income countries. A third benefit is the economical use of tissue, as molecular analysis might be performed later, and ample tissue is required.

Our study presents the first meta-analysis on this topic but is not without limitation. First, the predefined quality parameters could not always be identified in the included articles, negatively impacting our predefined subgroup analysis on quality. Second, for the objective of our study it would have been better if articles used uniform nomenclature and clear cut-offs for MMR IHC interpretations^68^. Furthermore, many articles had highly selected cohorts, which may have introduced bias. Finally, due to our liberal search-strategy a large series of articles was included.

Methodologically, all the included papers reported on the results of immunohistochemistry using all four MMR proteins. There was significant methodological heterogeneity regarding the MMR antibody clones and staining platforms used, reflecting real-world diversity of MMR testing. This variety in methodology will give some variability in MMR IHC staining intensity, but the impact on final interpretation of the MMR IHC is binary (negative/positive). We also allowed heterogeneity in tumor-types, as MMR status assignment is relevant and uniformly applied in a large array of solid tumours in daily practice. We decided to include all articles reporting on MMR IHC instead of only the articles with the aim of investigating the two vs four antibody approach, as this is a more realistic representation of clinical practice.

The statistical heterogeneity in the overall analysis was high, I^2^ = 87%. We tried to identify variables that accounted for the heterogeneity in subgroup analyses. In all our pre-defined subgroups heterogeneity was high (I^2^ > 50%), except for the subgroup analysis of the six articles with the aim of investigating the two vs four antibody approach. In this subgroup the statistical heterogeneity was 0% and no cases with non-dimeric staining patterns were observed, reflecting that a dedicated approach results in matching outcomes.

In conclusion, the results of our study support the use of the two-antibody testing algorithm, starting with PMS2 and MSH6, in clinical practice. Using this approach at least 98.9% of MMRd cases will be detected. However, our study suggests that the number of misclassified cases may be as low as zero when MMR IHC is interpreted by specialist pathologists. Educational sessions in which staining pattern pitfalls are discussed will remain important. Staining of all four antibodies should be performed when there is MMRd or any doubt about the interpretation of the staining patterns observed with the two-antibody testing algorithm.

## Supplementary information

Supplementary information, S1, S2, S5,S6 and S7

Supplementary information, S3

Supplementary information, S4

## Data Availability

All data generated and/or analyzed during the current study are included in this published article and its supplementary information files.
