# Supplementary material for: Validity of a two-antibody testing algorithm for mismatch repair deficiency testing in cancer; a systematic literature review and meta-analysis
Source: Mod Pathol. 2022 Sep 14;35(12):1775–83. doi: 10.1038/s41379-022-01149-w (PMC9708570; doi:10.1038/s41379-022-01149-w)

Supplementary information 1, Search strategy.

**PubMed**

<http://www.ncbi.nlm.nih.gov/pubmed?otool=leiden>

(("Immunohistochemistry"[Mesh] OR "immunohistochem*"[all fields] OR "immuno histochem*"[tw] OR "immune histochem*"[tw] OR "Immunocytochemistry"[tw] OR "Immunocytochem*"[tw] OR "Immunogold Silver Techniques"[tw] OR "Immunogold Silver Technic"[tw] OR "Immunogold Silver Technique"[tw] OR "Immunogold Technic"[tw] OR "Immunogold Technique"[tw] OR "Immunogold Techniques"[tw] OR "Immunohistocytochemistry"[tw] OR "Immunohistocytochem*"[tw] OR "Immunolabeling Technique"[tw] OR "Immunolabeling Techniques"[tw] OR "Enzyme Multiplied Immunoassay Technique"[tw] OR "Enzyme-Linked Immunosorbent Assay"[tw] OR "Enzyme-Linked Immunosorbent Assays"[tw] OR "Enzyme-Linked Immunospot Assay"[tw] OR "Enzyme-Linked Immunospot Assays"[tw] OR "Fluorescence Polarization Immunoassay"[tw] OR "Fluorescence Polarization Immunoassays"[tw] OR "Fluorescent Antibody Technic"[tw] OR "Fluorescent Antibody Technics"[tw] OR "Fluorescent Antibody Technique"[tw] OR "Fluorescent Antibody Techniques"[tw] OR "Fluoroimmunoassay"[tw] OR "Fluoroimmunoassays"[tw] OR "Immunoenzyme Technic"[tw] OR "Immunoenzyme Technics"[tw] OR "Immunoenzyme Technique"[tw] OR "Immunoenzyme Techniques"[tw]) AND ("DNA Mismatch Repair"[Mesh] OR "mismatch repair"[all fields] OR "mismatch repair*"[all fields] OR "mis match repair*"[all fields] OR "mismatch DNA repair"[all fields] OR (("mismatch*"[tw] OR "mis match*"[tw]) AND ("DNA"[tw] OR "gene"[tw] OR "genes"[tw]) AND "repair*"[tw])))

**Embase**

<http://ovidsp.ovid.com/ovidweb.cgi?T=JS&PAGE=main&MODE=ovid&D=oemezd>

((exp *"Immunohistochemistry"/ OR "immunohistochem*".ti,ab OR "immuno histochem*".ti,ab OR "immune histochem*".ti,ab OR "Immunocytochemistry".ti,ab OR "Immunocytochem*".ti,ab OR "Immunogold Silver Techniques".ti,ab OR "Immunogold Silver Technic".ti,ab OR "Immunogold Silver Technique".ti,ab OR "Immunogold Technic".ti,ab OR "Immunogold Technique".ti,ab OR "Immunogold Techniques".ti,ab OR "Immunohistocytochemistry".ti,ab OR "Immunohistocytochem*".ti,ab OR "Immunolabeling Technique".ti,ab OR "Immunolabeling Techniques".ti,ab OR "Enzyme Multiplied Immunoassay Technique".ti,ab OR "Enzyme-Linked Immunosorbent Assay".ti,ab OR "Enzyme-Linked Immunosorbent Assays".ti,ab OR "Enzyme-Linked Immunospot Assay".ti,ab OR "Enzyme-Linked Immunospot Assays".ti,ab OR "Fluorescence Polarization Immunoassay".ti,ab OR "Fluorescence Polarization Immunoassays".ti,ab OR "Fluorescent Antibody Technic".ti,ab OR "Fluorescent Antibody Technics".ti,ab OR "Fluorescent Antibody Technique".ti,ab OR "Fluorescent Antibody Techniques".ti,ab OR "Fluoroimmunoassay".ti,ab OR "Fluoroimmunoassays".ti,ab OR "Immunoenzyme Technic".ti,ab OR "Immunoenzyme Technics".ti,ab OR "Immunoenzyme Technique".ti,ab OR "Immunoenzyme Techniques".ti,ab) AND (*"Mismatch Repair"/ OR "mismatch repair".ti,ab OR "mismatch repair*".ti,ab OR "mis match repair*".ti,ab OR "mismatch DNA repair".ti,ab OR (("mismatch*".ti,ab OR "mis match*".ti,ab) ADJ4 ("DNA".ti,ab OR "gene".ti,ab OR "genes".ti,ab) ADJ4 "repair*".ti,ab)))

**Cochrane**

<https://www.cochranelibrary.com/advanced-search/search-manager>

(("Immunohistochemistry" OR "immunohistochem*" OR "immuno histochem*" OR "immune histochem*" OR "Immunocytochemistry" OR "Immunocytochem*" OR "Immunogold Silver Techniques" OR "Immunogold Silver Technic" OR "Immunogold Silver Technique" OR "Immunogold Technic" OR "Immunogold Technique" OR "Immunogold Techniques" OR "Immunohistocytochemistry" OR "Immunohistocytochem*" OR "Immunolabeling Technique" OR "Immunolabeling Techniques" OR "Enzyme Multiplied Immunoassay Technique" OR "Enzyme-Linked Immunosorbent Assay" OR "Enzyme-Linked Immunosorbent Assays" OR "Enzyme-Linked Immunospot Assay" OR "Enzyme-Linked Immunospot Assays" OR "Fluorescence Polarization Immunoassay" OR "Fluorescence Polarization Immunoassays" OR "Fluorescent Antibody Technic" OR "Fluorescent Antibody Technics" OR "Fluorescent Antibody Technique" OR "Fluorescent Antibody Techniq

ues" OR "Fluoroimmunoassay" OR "Fluoroimmunoassays" OR "Immunoenzyme Technic" OR "Immunoenzyme Technics" OR "Immunoenzyme Technique" OR "Immunoenzyme Techniques") AND ("Mismatch Repair" OR "mismatch repair" OR "mismatch repair*" OR "mis match repair*" OR "mismatch DNA repair" OR (("mismatch*" OR "mis match*") AND ("DNA" OR "gene" OR "genes") AND "repair")))

Supplementary information 2, R script.

# Main analysis

out <- metaprop(event = MMRDmissedBYtwostep, n =MMRd,

studlab = RefID, data=D, sm = "PAS",

prediction=TRUE, comb.random=TRUE,

method.tau = "EB")

out

# Forest plot main anlaysis

pdf('Forest plot all data mmrd.pdf', height = out$k/4.5, width=12)

forest.meta(out, prediction=TRUE, # layout = "RevMan5",

comb.fixed = FALSE, subgroup = cohortcancertype)

dev.off()

# subgroup analysis

out <- metaprop(event = MMRDmissedBYtwostep, n =MMRd,

studlab = RefID, data=D, sm = "PAS",

prediction=TRUE, comb.random=TRUE,

subset = subgroup == "soupgroupname",

method.tau = "EB")

out

#forest plot subgroup

pdf('Forest plot subgroup gyn.pdf', height = 5+out$k/4.5, width=12)

forest.meta(out, prediction=TRUE, # layout = "RevMan5",

comb.fixed = FALSE,subgroup = subgroup)

dev.off()

Supplementary information 5, Forest plot All included articles
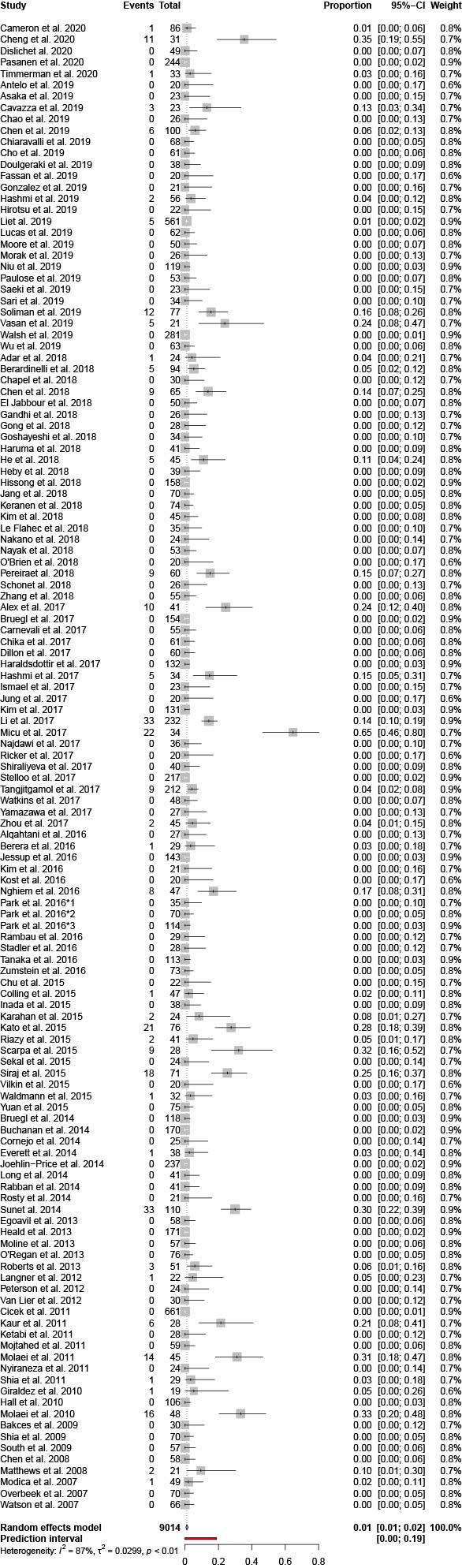


Supplementary information 6, Forest plot articles with the aim of investigating the two-antibody testing-algorithm.


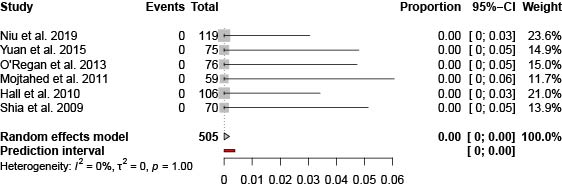


Supplementary information 7, Forest plot Dermatology oriented articles


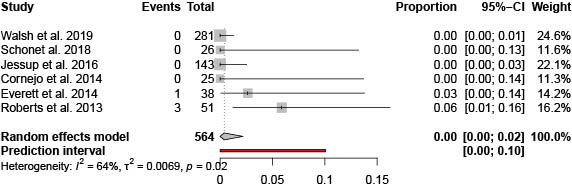

Supplement: Supplementary file 1 — Supplementary information, S1, S2, S5,S6 and S7 [file 41379_2022_1149_MOESM1_ESM.doc]
